# When less is more: validating a brief scale to rate interprofessional team competencies

**DOI:** 10.1080/10872981.2017.1314751

**Published:** 2017-05-05

**Authors:** Désirée A. Lie, Regina Richter-Lagha, Christopher P. Forest, Anne Walsh, Kevin Lohenry

**Affiliations:** ^a^ Department of Family Medicine, Keck School of Medicine of the University of Southern California, CA, USA

**Keywords:** Interprofessional education, team behaviors, assessment, team objective structured clinical encounter, validation, rating scale

## Abstract

**Background**: There is a need for validated and easy-to-apply behavior-based tools for assessing interprofessional team competencies in clinical settings. The seven-item observer-based Modified McMaster-Ottawa scale was developed for the Team Objective Structured Clinical Encounter (TOSCE) to assess individual and team performance in interprofessional patient encounters.

**Objective**: We aimed to improve scale usability for clinical settings by reducing item numbers while maintaining generalizability; and to explore the minimum number of observed cases required to achieve modest generalizability for giving feedback.

**Design**: We administered a two-station TOSCE in April 2016 to 63 students split into 16 newly-formed teams, each consisting of four professions. The stations were of similar difficulty. We trained sixteen faculty to rate two teams each. We examined individual and team performance scores using generalizability (G) theory and principal component analysis (PCA).

**Results**: The seven-item scale shows modest generalizability (.75) with individual scores. PCA revealed multicollinearity and singularity among scale items and we identified three potential items for removal. Reducing items for individual scores from seven to four (measuring Collaboration, Roles, Patient/Family-centeredness, and Conflict Management) changed scale generalizability from .75 to .73. Performance assessment with two cases is associated with reasonable generalizability (.73). Students in newly-formed interprofessional teams show a learning curve after one patient encounter. Team scores from a two-station TOSCE demonstrate low generalizability whether the scale consisted of four (.53) or seven items (.55).

**Conclusion**: The four-item Modified McMaster-Ottawa scale for assessing individual performance in interprofessional teams retains the generalizability and validity of the seven-item scale. Observation of students in teams interacting with two different patients provides reasonably reliable ratings for giving feedback. The four-item scale has potential for assessing individual student skills and the impact of IPE curricula in clinical practice settings.

**Abbreviations:** IPE: Interprofessional education; SP: Standardized patient; TOSCE: Team objective structured clinical encounter

## Introduction

Team-based care has been associated with improved healthcare outcomes [1,2] and patient satisfaction [3]. Interprofessional Education (IPE) is recognized as a pathway to prepare students for future interprofessional practice and collaboration [4–9]. Many accreditation bodies now include IPE as a training requirement [10]. Validated tools for assessing teamwork competencies are needed to effectively translate IPE teaching to practice [11].

**Figure 1.**
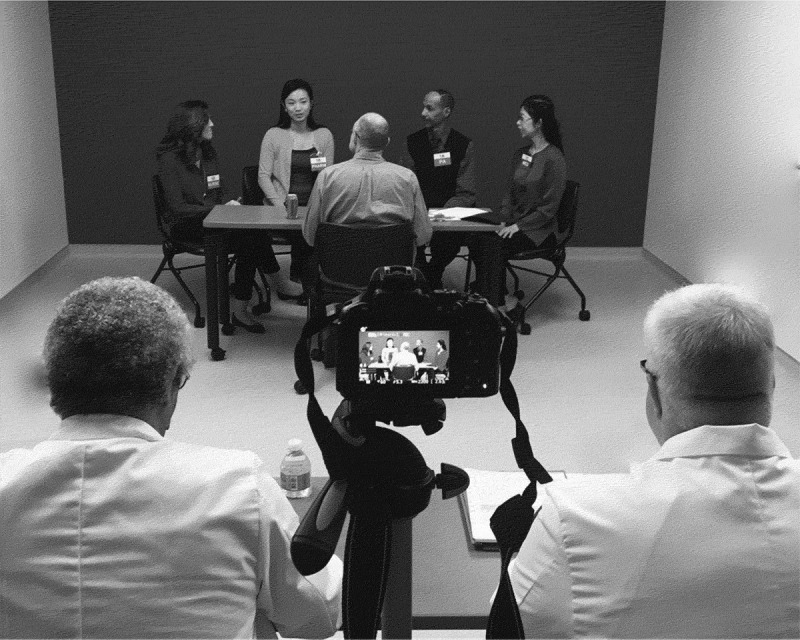
Team Objective Structured Clinical Encounter room setup showing camera setup with faculty raters in the foreground and students positioned in the far side of the room, Keck School of Medicine of the ​University of Southern California, 2016.

The Kirkpatrick framework [12] recommends demonstration of behavior change as part of competency-based learning, yet there is a dearth of behavioral measures beyond self-report in IPE [13]. A recent systematic review of teamwork assessment tools in internal medicine [14] concluded that published tools are supported by evidence of validity, but most are based on subjective reports of satisfaction or attitude. A systematic review of allied health programs found a lack of assessment tools of observed behaviors [15], while another [16] identified only four observer-rated checklists from among 48 measures of team performance in primary care. A review of assessment tools for interprofessional collaboration in undergraduate medical education [11] identified from among 64 tools only two direct observation scales addressing teamwork competencies. The Teamwork Mini-Clinical Evaluation Exercise (T-MEX) is a workplace-based seven-item scale that measures six observed behaviors in the domains of supportive relationships, self-awareness/responsibility, and safe communication [17]. It is designed for one health profession (medicine) and requires multiple observations by different raters [18]. The Communication and Teamwork Skills (CATS) [19,20] scale assesses teamwork practice behaviors in communication, coordination, cooperation and situational awareness, requires rater training and has been tested on three health professions. However, with 18 items, it is too lengthy to apply to multiple individuals during one team-patient observation.

A brief validated observer-based scale is needed to efficiently assess team members and the team’s performance in a patient encounter [15,21]. The McMaster-Ottawa scale [22–25] is a seven-item, nine-point scale developed for rating individual and team behaviors in a standardized setting of a Team Objective Structured Clinical Encounter (TOSCE). The scale addresses six interprofessional competencies of communication, collaboration, roles and responsibilities, patient/family-centered approach, conflict management and teamwork, congruent with established IPE competencies [4,7], with an additional global score. The face and content validity of the scale were reported in several studies [23,24,26]. The scale purports to evaluate individual and team performance in clinical settings [25,27,28]. The scale’s advantage is that the team behaviors assessed are not profession-specific. The scale was modified from 9 to 3 points with descriptive behavioral anchors [28] for ease of rater training, without sacrificing reliability, and found to be feasible to apply in a standardized patient (SP) setting. However, the modified scale remains a challenge to apply in busy settings where faculty are often limited to 30 minutes or less to simultaneously observe several students in a team encounter.

We aimed to refine the modified scale to increase its usability for clinical settings. We focused on two issues: (1) the length (item number) of the scale, and (2) the number of cases necessary to achieve modest levels of generalizability. We conducted a study in a TOSCE setting to control case difficulty and faculty scoring. Based on overlap in the constructs measured (for example, between ‘communication’ and ‘teamwork’) we hypothesized that the scale can be reduced from seven to four items and still maintain reasonable levels of generalizability, or reliability, and validity. We also explored whether ratings from two stations or cases were sufficiently generalizable to provide feedback. The study received institutional board review approval (IRB#: HS-12-00223).

## Methods

### Study setting

Our study was conducted at the University of Southern California, Los Angeles, USA, and involved four health professions (Physician Assistant, Pharmacy, Occupational Therapy, and Nursing).

### Study participants – students

We recruited, by email, volunteer students from the four health professions programs. No predetermined criteria were used other than willingness to participate on a Saturday morning and comfort seeing patients. Students were informed that the TOSCE was a formative interprofessional experience, ratings would be de-identified, and no results would be shared with their supervisors. Participants were given $25 to cover transport costs.

### Study participants – faculty raters

We recruited 16 volunteer faculty raters from the same four professions by enlisting their participation via an email listserv of an IPE committee. The criterion was previous experience evaluating students in clinical settings. Experience with IPE or the TOSCE was not required.

### TOSCE design

We designed a two-station TOSCE so that each team of four students would work with two different SPs in succession. Each student would receive individual ratings from the same two faculty for each station. We assigned a pair of faculty raters to each team because our previous work [29] indicated that two faculty were needed to optimize reliability for ‘below expected’ ratings. Each faculty pair rated two different teams in succession. Students were assigned to their teams just before the TOSCE. Students in each team did not know one another. For each TOSCE station, the student team was provided with a case scenario, instructed to assess the SP and prepare a plan for presentation to an attending. The two stations were designed at a similar level of difficulty using a common template, to minimize the impact of sequence of exposure on team performance. Each station (one an SP with chronic obstructive airway disease, the other an SP with diabetes) lasted 25 minutes: five minutes for a team pre-huddle [30], 15 minutes with the SP, and five minutes for a post-huddle. Raters were present for all 25 minutes of performance and were given five minutes between stations to complete their rating forms (see Figure 1 for room setup).

### Rater training

One week prior to the TOSCE, faculty raters received an email link to a standardized training video and the scale [31]. They were asked to review the video and complete the ratings on the actor students (each of whom performed at a different level) and the team portrayed in the video. They then received one hour of in-person group training using the same video, just before being assigned to their student team. We utilized principles from frame-of-reference training [32,33] and rater error training [32]. Faculty trainers (CF, AW, KL) asked for independent ratings, then used a discussion format focused on items with greatest rating discrepancies, to achieve consensus among raters.

### Data collection

Each faculty independently completed paper rating forms [29,31] for students and teams. Student and team station scores were later constructed by averaging all seven items by the rater. Ratings for each student and team were entered into Excel and analyzed using SPSS, version 23 (IBM SPSS Statistics 23.0 IBM Corp. IBM SPSS Statistics for Windows, Version 23.0: IBM Corp) and GENOVA [34].

### Data analysis

We examined score differences within each pair of faculty raters to determine inter-rater reliability. We examined student and team scores, using descriptive statistics and *t-*tests to compare scores. We also investigated the possibility of a learning curve effect, examining for significant improvement between the first and second stations.

We performed a generalizability study (g-study), using GENOVA, to determine the minimum number of scale items and stations necessary to maintain modest levels of generalizability or reliability. Generalizability theory posits that variation in performance scores can be deconstructed into variation attributable to actual student (or team) ability and error [35]. By better understanding the contributions of each of these sources to overall variation in scores, we can determine methods for improving measurement design. In this case, student performance scores were deconstructed into person (*p*) variation, or variation based on differences in examinee ability, and error variation attributable to differences between station (*s*), and item (*i)* as well as the interaction between person and station (*ps*) and person and item (*pi*). While rater (*r*) could also be a source of possible error variation, in this study, raters were nested within each station, meaning error variation attributable to the rater could not be distinguished from error variation attributable to the station. Based on results from our previous study [25], we made a concerted effort to train faculty to ensure standardization of ratings; therefore, for the purpose of this study, variation in scores attributable to station is assumed to be a result of differences in station difficulty (which we controlled for), not rater differences. The generalizability (or reliability), of student scores, represents the proportion of variance in scores attributable to differences in ability (*p*) versus the proportion of variance attributable to these other sources of error (like station and item and their interactions), also known as facets. While estimated differently from coefficient alpha, the generalizability coefficient is considered conceptually analogous, meaning values between .70 and. 80 are considered acceptable levels of reliability.

Based on findings of the g-study, we then conducted a principal components analysis (PCA), using SPSS, to determine what items, if any, would be good candidates for removal from the scale.

## Results

### Participants (Table 1)

Sixty-eight students responded to the invitation to participate and 63 participated. Fifteen of 16 teams had four team members and one team had three members. Sixteen faculty from the four professions volunteered to be raters and received one hour of face-to-face standardized rater training [31]. Students and faculty were predominantly female. Thirty-three of 63 students reported prior IPE exposure.

**Table 1.** Demographics of students and faculty participating in Team Objective Structured Clinical Encounter Keck School of Medicine of the University of Southern California, 2016.

Student profession	Number of students (N = 63)	Preclinical N	Age group		Female N	Received prior IPE* training N
			≥31 years N	<31 years N		
Nursing	15	0	10	5	14	4
Occupational therapy	16	10	15	1	14	13
Pharmacy	16	14	15	1	11	7
Physician assistant	16	16	14	2	11	9
Faculty profession	Number (N = 16)		Mean years in education		Female N	
Nursing	3		11.5		3	
Occupational therapy	4		2.4		4	
Pharmacy	4		1.0		3	
Physician assistant	5		7.4		5	

*IPE: Interprofessional Education

### TOSCE administration

The TOSCE was administered in the planned timeframe of four hours. Each pair of faculty rated two student teams performing sequentially at the two stations. All faculty submitted their ratings.

### Student and team performance scores

There were no significant differences between individual student and team scores within each faculty rater pair (Table 2). Based on this finding reflecting high inter-rater reliability, we constructed student and team scores using the average of the two raters in each station.

**Table 2.** Differences between team and student scores by faculty rater and by station, Keck School of Medicine of the University of Southern California, 2016.

	Team *N *= 16				Student *N *= 63			
	Faculty rater 1	Faculty rater 2			Faculty rater 1	Faculty rater 2		
Station	*M (SD)*		*M_diff_*	*t*-test	*M (SD)*		*M_diff_*	*t*-test
Station 1	2.0 (0.5)	2.1 (0.6)	−0.1	−0.78	1.9 (0.6)	2.1 (0.5)	−0.1	−1.83
Station 2	2.1 (0.5)	2.2 (0.5)	−0.1	−0.59	2.1 (0.5)	2.2 (0.5)	0.0	0.99
Total	2.1 (0.5)	2.2 (0.4)	−0.1	−0.87	2.0 (0.5)	2.1 (0.4)	−0.1	−1.70

There were no differences in student scores by gender, age, profession, or training stage (pre-clinical vs. clinical). There was a statistically significant difference in performance between students who reported any prior interprofessional experience compared with those who reported none, in both station 1, *t*(61) = −2.78, *p* = .007, *d* = 0.71, and station 2, *t*(61) = −2.23, *p* = .029, *d* = 0.55. Although score differences between professions were not significant, nursing students, who more frequently reported no prior interprofessional experiences, on average scored the lowest in both stations. A paired samples *t*-test indicated that student scores significantly improved in the second station, *t*(62) = −2.73, *p* = .008, *d* = 0.34, suggesting a possible learning curve effect.

### Number of scale items (individual and team scores)

The seven-item scale shows modest generalizability (.75) with individual scores for two stations. We used g-theory to examine the proportion of variance in individual scores attributable to an item and the interaction between person and item, to determine the number of items necessary to ensure adequate generalizability of scores.

Individual variance components and estimates of generalizability of scores by person or student (*p*) x station (*s*) x item (*i*) (Table 3) demonstrated that over 70% of the total variance in student performance scores for the seven items was attributable to systematic differences between students. Averaged faculty ratings of students on the seven items in each station indicated that only about 2% of variation in student scores were attributable to station (0.01625), indicating similar levels of difficulty between the two stations. Almost 2% of variation in student scores was attributable to item (0.01428), indicating no item on the scale was more or less difficult than another. A larger proportion (about 19%) of the score variance was attributable to the interaction between student and station (0.14551) suggesting that the relative standing of students varied from station to station. Almost 2% of the variation in scores was attributable to the interaction between student and item (0.01272), meaning that the relative standing of students did not vary from item to item. Subsequent decision studies indicate that a scale consisting of five items to score individual student performance would yield modest generalizability on a two-station TOSCE (.74), while a scale consisting of four items would yield similar levels of generalizability (.73).

**Table 3.** Estimated variance components for student and team performance scores based on two-station team objective structured clinical encounter, Keck School of Medicine of the University of Southern California, 2016.

Source of variance	df^a^	2 stations 1 item^b^	2 stations 2 items^b^	2 stations 3 items^b^	2 stations 4 items^b^	2 stations 5 items^b^	2 stations 6 items^b^	2 stations 7 items^b^	4 stations 4 items^b^	4 stations 5 items^b^	8 stations 4 items^b^	8 stations 5 items^b^
		Individual student										
Student (*p*)	62	0.54588 (52.21)	0.54588 (62.27)	0.54588 (66.55)	0.54588 (68.92)	0.54588 (70.42)	0.54588 (71.45)	0.54588 (72.22)	0.54588 (79.76)	0.54588 (80.34)	0.54588 (84.92)	0.54588 (86.43)
Station (*s*)	1	0.01625 (1.55)	0.01625 (1.85)	0.01625 (1.98)	0.01625 (2.05)	0.01625 (2.10)	0.01625 (2.13)	0.01625 (2.15)	0.00812 (1.19)	0.00812 (1.20)	0.00406 (0.63)	0.00406 (0.64)
Item (*i*)	6	0.09996 (9.56)	0.04998 (5.70)	0.03332 (4.06)	0.02499 (3.15)	0.01999 (2.58)	0.01666 (2.18)	0.01428 (1.89)	0.02499 (3.65)	0.01999 (2.94)	0.02490 (3.87)	0.01999 (3.17)
*ps*	62	0.14551 (13.92)	0.14551 (16.60)	0.14551 (17.74)	0.14551 (18.37)	0.14551 (18.77)	0.14551 (19.05)	0.14551 (19.25)	0.07275 (10.63)	0.07275 (10.71)	0.03638 (5.66)	0.03638 (5.76)
*pi*	372	0.08901 (8.51)	0.04450 (5.08)	0.02967 (3.62)	0.02225 (2.80)	0.01780 (2.30)	0.01483 (1.94)	0.01272 (1.68)	0.02225 (3.25)	0.01780 (2.62)	0.02225 (3.46)	0.01780 (2.82)
*si*	6	0.00169 (0.16)	0.00085 (0.10)	0.00056 (00.07)	0.00042 (0.05)	0.00034 (0.04)	0.00028 (0.04)	0.00024 (0.03)	0.00021 (0.00)	0.00017 (0.03)	0.00011 (0.02)	0.00008 (0.01)
*psi,e*	372	0.14721 (14.08)	0.07361 (8.40)	0.04907 (5.98)	0.03680 (4.65)	0.02944 (3.80)	0.02454 (3.21)	0.02103 (2.78)	0.01840 (2.69)	0.01472 (2.17)	0.00920 (1.43)	0.00736 (1.17)
*Generalizability*		.59	.67	.71	.73	.74	.75	.75	.83	.84	.89	.90
		Team										
Team (*t*)	15	0.30933 (31.56)	0.30933 (41.18)	0.30933 (45.83)	0.30933 (48.57)	0.30933 (50.38)	0.30933 (53.55)	0.30933 (52.62)	0.30933 (61.70)	0.30933 (63.88)	0.30933 (71.34)	0.30933 (73.76)
Station (*s*)	1	0.00536 (0.55)	0.00536 (0.72)	0.00536 (0.79)	0.00536 (0.84)	0.00536 (0.87)	0.00536 (0.91)	0.00536 (0.91)	0.00268 (0.53)	0.00268 (0.55)	0.00134 (0.31)	0.00134 (0.32)
Item (*i*)	6	0.17465 (17.82)	0.08733 (11.63)	0.05822 (8.63)	0.04366 (6.86)	0.03493 (5.69)	0.02911 (4.94)	0.02495 (4.24)	0.04366 (8.71)	0.03493 (7.21)	0.04366 (10.07)	0.03493 (8.33)
*ts*	15	0.20774 (21.20)	0.20774 (27.65)	0.20774 (30.78)	0.20774 (32.62)	0.20774 (33.84)	0.20774 (35.29)	0.20774 (34.34)	0.10387 (20.72)	0.10387 (21.45)	0.05193 (11.98)	0.05193 (12.38)
*ti*	90	0.05139 (5.24)	0.02569 (3.42)	0.01713 (2.54)	0.01285 (2.02)	0.01028 (1.67)	0.00856 (1.45)	0.00734 (1.25)	0.01285 (2.56)	0.01028 (2.12)	0.01285 (2.96)	0.01028 (2.45)
*ti*	6	0.00000 (0.00)	0.00000 (0.00)	0.00000 (0.00)	0.00000 (0.00)	0.00000 (0.00)	0.00000 (0.00)	0.00000 (0.00)	0.00000 (0.00)	0.00000 (0.00)	0.00000 (0.00)	0.00000 (0.00)
*tsi,e*	90	0.23155 (23.63)	0.11577 (15.41)	0.07718 (11.43)	0.05789 (9.09)	0.04631 (7.54)	0.02859 (4.86)	0.03308 (5.63)	0.02894 (5.77)	0.02315 (4.78)	0.01447 (3.34)	0.01158 (2.76)
*Generalizability*	.39	.39	.47	.51	.53	.54	.55	.55	.68	.69	.80	.81

^a^Degrees of freedom

^b^Variance component (% of total variance)

The variance by team (*t*) x station (*s*) x item (*i*) using g-study is shown in Table 3. A large proportion (34.34%) of error variance was attributable to the interaction of team and station (0.20774), meaning a team that scored relatively high in one station did not necessarily score relatively high in the other station. As a result, analysis of team scores from a two-station TOSCE revealed low generalizability, whether the scale consisted of four (.53) or seven items (.55).

We then conducted a PCA to determine which items, if any, would make good candidates for removal when scoring individual performance. Examination of correlations between items, based on the average performance across the two stations by item, indicated strong, positive, statistically significant relationships (Table 4), indicating possible multicollinearity. Both the Global item and the Communication item scores had high inter-item correlation with all other items. The correlation between the Communication and Global items was strong (*r *= .90) suggesting singularity. The determinant of the correlation matrix was 0.000, again indicating multicollinearity. As a result, the Global item was removed, resulting in a determinant of 0.005. The Kaiser-Meyer-Olkin measure indicated sampling adequacy, *KMO* = .90, and Bartlett’s Test of Sphericity, *Χ^2^* (21) = 316.68, *p* < .001, indicated that PCA was appropriate given the data. Extraction of factors yielded one factor that explained 76.67% of the variance in scores. We found communalities indicating large proportions of common variance in the data structure by item (Table 5). Given the high correlation between Communication and other items, we also examined the factor structure when both the Communication and Global items were removed. Results indicated sampling adequacy, *KMO* = .87, while Bartlett’s Test of Sphericity, *Χ^2^* (15) = 221.74, *p* < .001, indicated the appropriateness of PCA. Extraction of factors yielded a one-factor solution that explained 75.69% of the variance in scores. A further examination investigated the removal of the Teamwork item, which also correlated strongly with other items, from the analysis. Results indicated sampling adequacy, *KMO* = .80, while Bartlett’s Test of Sphericity, *Χ^2^* (6) = 139.56, *p* < .001, indicated the appropriateness of PCA. Extraction of factors yielded a one-factor solution that explained 74.60% of the variance in individual scores.

**Table 4.** Inter-item correlation matrix for the modified McMaster-Ottawa scale (individual student scores) when applied to two stations, Keck School of Medicine of the University of Southern California, 2016.

Item	Communication	Collaboration	Roles	Patient-centered	Conflict	Team
Collaboration	.80					
Roles	.69	.77				
Patient-centered	.70	.66	.56			
Conflict	.80	.69	.61	.67		
Team	.83	.79	.77	.71	.72	
Global	.90	.84	.77	.81	.78	.86

All inter-item correlations were statistically significant, *p* < .001

**Table 5.** Communalities of items after extraction for the modified McMaster-Ottawa scale (individual student scores) when applied to two stations, Keck School of Medicine of the University of Southern California, 2016.

Item	6-item scale	5-item scale	4-item scale
Communication	.85	–	–
Collaboration	.81	.82	.83
Roles	.70	.73	.73
Patient-centered	.66	.67	.69
Conflict	.73	.72	.74
Team	.85	.85	–

### Number of stations (individual and team scores)

For a two-station TOSCE using a four-item scale, nearly 70% of the total variance in individual student scores would be attributable to systematic differences between students (Table 4). As discussed, averaged faculty ratings of students on the items in each station indicated that only 2% of variation was attributable to station (0.01625). About 18% of score variance was attributable to the interaction between person or student, and station (0.14551) suggesting that the relative standing of students varied from station to station. By changing the number of stations (Table 5), we can reduce the error variance attributable to station (*s)* and person-by-station (*ps*), thereby improving the generalizability of scores. For example, an eight-station TOSCE would dramatically reduce the estimated proportion of error variance attributable to the interaction between person and station (almost 6%, or 0.03638), increasing generalizability for individual scores to .90.

A g-study examining the variance in scores by team (*t*) x station (*s*) x item (*i*) indicated low generalizability for team scores (data not shown). A four-station TOSCE would achieve modest levels of generalizability (.68) of team scores. An eight-station TOSCE would achieve higher levels of generalizability (.80).

## Discussion

We applied the Modified seven-item McMaster-Ottawa scale to rate new student teams in a two-station TOSCE. Our purpose was to ‘translate’ the scale [23,24,26,29] to use in clinical settings where faculty are challenged by limited time for observing and assessing several students at once. We optimized rater reliability by rigorous training, evidenced by high inter-rater reliability between raters. This finding affirms the importance of rater training before applying the scale [36]. We found that the four-item scale for scoring individual students in the competencies of Collaboration, Roles, Patient/Family-centered Care, and Conflict Management (see Appendix), retains the generalizability of the seven-item scale. PCA supports the removal the Global, Communication, and Teamwork items. We also found that the number of stations required to achieve modest levels of generalizability of student scores was small, likely due to minimizing error in scores attributable to station differences and providing standardized rater training. This implies that in practice settings, feedback to students based on two patient encounters of similar difficulty would be reliable. This is an important advantage compared with the multiple observations required of other scales such as the T-MEX [14].

Our finding that students who reported prior IPE experience scored higher compared to students reporting none, confirms observations from another study [37] and provides support for the efficacy of IPE for improving team behaviors. We speculate that the abbreviated scale is sensitive to performance differences between groups and can potentially function to evaluate the impact of new IPE curricula.

Team scores, however, demonstrate low generalizability, regardless of the number of scale items or stations. This may be due to the inherent variability one can expect when four students work with one patient. For example, a rater may assess a team as ‘high-performing’ when only two of four students show excellent ‘collaboration’ while another rater expects all four students to demonstrate excellence in collaboration before giving the team a high score on the same competency. Therefore, in our opinion, team scores for this scale are not appropriate for high-stakes summative assessment.

TOSCEs may not be a preferred method for summative assessment of interprofessional team competencies because of the challenges of expense and logistics to coordinate across professional schools or programs. Our finding that students show improved performance after working together with only one patient suggests that a multi-station TOSCE may be better suited for training to prepare for practice than for summative evaluation. We concur with recent recommendations of a seven-university Australian consortium [38] which developed a 10- and 11-item (3-point) individual Teamwork Observation and Feedback Tool (yet to be validated), to focus on formative rather than summative assessment of individual teamwork behaviors.

Our brief scale offers busy clinicians the opportunity to assess individual students working on teams and addresses an outstanding challenge facing IPE educators: that of limited faculty resource and IPE training sites [30,38]. Assessing interprofessional teams in patient settings is an emerging ‘real world’ approach to evaluate the impact of IPE curricula. Practical tools for assessing curricular effectiveness are needed at these sites [15,39–41]. The four-item scale enjoys the advantages (not shared by scales such as the CATS [17,18,20]) of being applicable to different professions and having an accessible published faculty rater training resource [31]. Faculty can complete individual assessments of multiple students in a team within 30 minutes of observation, while providing teaching and patient care. The finding that scale generalizability for individuals is reasonable with only two observed cases would ease the burden of student evaluation.

Our study has several strengths. Our hypothesis is rooted in an established theoretical framework of competency-based assessment [12,13]. We standardized faculty training, team composition, and case difficulty to minimize variables to focus on scale generalizability. Our student teams represented diverse professions. We utilized two stations to examine the effect of practice on performance at a second station. We maximized the yield of the TOSCE by using g-theory. Our study also has limitations. Our setting is standardized, and the feasibility of faculty observing and rating a team of four students in a busy clinical setting is yet to be tested. We involved only four professions. However, prior studies have already suggested that the scale is applicable to other health professions [23,24,26,29].

## Conclusion

The brief four-item Modified McMaster-Ottawa scale assessing the competencies of Collaboration, Roles, Patient/Family-centeredness, and Conflict Management offers a feasible and practical option for assessing team competencies in clinical settings. Performance assessment with two cases is associated with reasonable generalizability (.73) that allows for individual feedback. Students in newly-formed interprofessional teams show a learning curve after one patient encounter. Team scores demonstrate low generalizability regardless of item and station number. We recommend field testing to further examine the utility and psychometric properties of the four-item scale for evaluating student and IPE curriculum performance in clinics.

## Previous presentations

Presented as a research abstract at the Innovations in Medical Education meeting, San Gabriel, CA, USA, on 26 February, 2017

## Appendix: Brief (four-item) modified McMaster-Ottawa Scale (for individual rating) with anchors, Keck School of Medicine of the University of Southern California, 2016

Observe each team member during the huddles and patient encounter. Using the three-point scale, assess *each* member’s demonstration of behaviors for each of the four competencies. Please score all behaviors. Do not leave any item blank unless instructed to do so.

**Table:**

COMPETENCIES	INDIVIDUAL RATING		
	Below Expected	At Expected	Above Expected
**Collaboration** Establishes collaborative relationships Integration of perspectives Ensures shared information	1	2	3
**Roles and Responsibilities** Describes roles and responsibilities Shares knowledge with others Accepts accountability	1	2	3
**Collaborative Patient/Family Centered Approach** Seeks input from patient and family Shares with patient and family Advocates for patient and family	1	2	3
**Conflict Management /Resolution** Demonstrates active listening Respectful of different perspectives Works with others to prevent conflict	1	2	3

### Individual Behavioral Anchors for Competencies

**Collaboration: *Above Expected***: Incorporates information provided by others; ensures information is disseminated to entire team. ***At Expected***: Uses information provided by team members. ***Below Expected***: Does not use information provided by members.

**Roles/Responsibilities: *Above Expected***: Shows initiative describing own role/scope; asks for and clarifies members’ roles/responsibilities; describes contributions of other professions to team’s task; uses evidence-based practice to inform actions; clearly describes the rationale and takes responsibility for own challenging or blameworthy actions. ***At Expected***: Articulates own role when asked; inquires about team members’ roles/responsibilities; shares evidence-based practice; describes actions. ***Below Expected***: Does not ask roles/responsibilities of others; does not take ownership of decisions; if challenged, vague in description of actions.

**Collaborative Patient/Family Centered Approach: *Above Expected***: Provides patient/family with options for care including pros/cons; actively summarizes and attempts to incorporate family members’ views in care plans. ***At Expected***: Listens/solicits family members’ views; provides patient/family with options for care; articulates these needs to the team. ***Below Expected***: Ignores family’s or patient’s views/needs, fails to provide options for care.

**Conflict Management Resolution: *Above Expected***: Seeks harmony by listening respectfully to all; acknowledges and processes conflict; initiates resolution, seeks consensus, respects differing opinions; develops common agreement. ***At Expected***: Listens to team members, asks for feedback, recognizes conflict but does not develop common agreement. ***Below Expected***: Ignores and interrupts team members, avoids acknowledging conflict.
